# Intraindividual Double Burden of Malnutrition in Chinese Children and Adolescents Aged 6–17 Years: Evidence from the China Health and Nutrition Survey 2015

**DOI:** 10.3390/nu13093097

**Published:** 2021-09-03

**Authors:** Xiao Hu, Hongru Jiang, Huijun Wang, Bing Zhang, Jiguo Zhang, Xiaofang Jia, Liusen Wang, Zhihong Wang, Gangqiang Ding

**Affiliations:** Department of Public Nutrition, National Institute for Nutrition and Health, Chinese Center for Disease Control and Prevention, Beijing 100050, China; 17862957224@163.com (X.H.); jianghr@ninh.chinacdc.cn (H.J.); wanghj@ninh.chinacdc.cn (H.W.); zzhangb327@aliyun.com (B.Z.); zhangjg@ninh.chinacdc.cn (J.Z.); jiaxf@ninh.chinacdc.cn (X.J.); wangls@ninh.chinacdc.cn (L.W.); dinggq@chinacdc.cn (G.D.)

**Keywords:** malnutrition, double burden, micronutrient inadequacies, overweight/obesity, children and adolescents, China

## Abstract

Studies on the intraindividual double burden of malnutrition (DBM) among Chinese children and adolescents were lacking. This study aimed to analyze the prevalence of intraindividual DBM defined as the coexistence of overweight/obesity and dietary micronutrient intake insufficiency and investigate dietary micronutrient intake in Chinese children and adolescents. Using data from the 2015 China Health and Nutrition Survey (CHNS), 1555 children and adolescents aged 6 to 17 years were selected as the subjects. We referred to China Food Composition to calculate the intakes of 11 selected dietary micronutrients from diet data collected by consecutive three days of 24 h recalls combined with household weighing of seasonings. We used the Chinese estimated average requirement (EARs) as a cutoff to define the dietary micronutrients deficiency, and applied the body-mass-index-for-age Z-scores (BAZ) of World Health Organization (WHO) child growth standards to define the category of body weight. Among the subjects in present study, the prevalence of overweight and obesity was 15.43% and 11.06%, respectively, and 26.24% of the subjects had undergone intraindividual DBM. The results suggest that the prevalence of intraindividual DBM and dietary micronutrients deficiency in Chinese children and adolescents is high.

## 1. Introduction

Malnutrition persists at unacceptably high levels on a global scale; one in every nine people in the world is hungry, and one in every three is overweight or obese [1]. More and more countries experience the double burden of malnutrition (DBM), which is characterized by the coexistence of undernutrition (i.e., micronutrient deficiencies, underweight and childhood stunting and wasting) alongside overweight, obesity, or other diet-related noncommunicable diseases [2]. In 2016, overweight and obesity were increasing among Tajik women aged 15–49 years, and the deficiency of iron, vitamin A, folate and iodine was serious [3]. Another study highlighted the DBM of Vietnam children in 2011 with the dietary intake insufficiency of iron, vitamin A, vitamin B1 and vitamin C, and the prevalence of overweight, obesity and underweight [4].

With rapid development, China has experienced a seismic shift from a high prevalence of underweight to increasing overweight and obesity over the past few decades [5,6,7], the prevalence of overweight/obesity increased from 5.0% and 1.7% in 1991–1995 to 11.7% and 6.8% in 2011–2015, respectively [1,8,9]. The dietary changes are enormous in China [10,11]. Apart from the concerning condition of overweight and obesity, dietary micronutrient intake deficiency in children and adolescents is also a problem. An estimate as high as 96% of 4–17-year-old participants had inadequate dietary intake of calcium, and the dietary intake of iron, zinc, selenium, vitamin A, thiamine, riboflavin and vitamin C was insufficient to varying degrees [12].

Several studies have revealed the desperate state of micronutrient inadequacy among children and adolescents [12,13,14]. It is generally wrongly thought that obese subjects are just in over-nutrition and it is impossible for them to have micronutrient deficiency. The prevalence of DBM at the intraindividual level with respect to micronutrient deficiencies was less well documented. Efforts to monitor the prevalence of DBM at an intraindividual level are necessary to facilitate children’s health precisely by providing related information. To achieve these goals, the objectives of this study were to (1) describe the prevalence and distribution of intraindividual DBM in Chinese aged 6 to 17 years, defined as coexistence of overweight/obesity and dietary micronutrient intakes deficiencies; (2) estimate the selected dietary micronutrient intakes and further identify differences in dietary micronutrient status between different body weight groups.

## 2. Materials and Methods

### 2.1. Study Population

This survey was based on the data from the China Health and Nutrition Survey (CHNS), which is an ongoing large-scale, longitudinal household survey of Chinese population. The CHNS covered 9 provinces in the first eight rounds during the period of 1989–2009. In 2015, the 10th round of the CHNS has covered 15 provinces and cities. It used a multistage, stratified, random cluster sampling design to select communities, and the survey included individual, household and community materials. Detailed information about the background, aims, design and methods of the CHNS have been illustrated in elsewhere [15].

Our analysis used the 10th round survey of the CHNS in 2015. We selected the participants aged 6 to 17 years who had complete records of diets, anthropometric, demographic and socioeconomic materials, resulting in a total sample of 1555 (806 boys and 749 girls) subjects. All the participants’ parents/guardians provided written informed consent for their children’s participation in the survey. The CHNS was approved by the institutional review committees of the University of North Carolina at Chapel Hill and the National Institute for Nutrition and Health, Chinese Center for Disease Control and Prevention (No. 201524).

### 2.2. Assessment of Dietary Micronutrient Intakes

The daily dietary intake information was collected by three consecutive days (two weekdays and one weekend day) recalls for each subject. For children under 12 who eat at home, the mother or caregiver responsible for food preparation was asked to recall the children’s food consumption. The trained health workers interviewed the participants face-to-face every day during the survey period to collect all data on food consumption at home and eating out during the preceding 24 h. The consumption of edible oil and seasonings in three days was collected by the household weighing accounting method, and the consumption of edible oil and seasonings was allocated to individuals according to the proportion of individual energy consumption in the family. The China Food Composition Tables published in 2009 [16] was referred to code and calculate the three-day average intakes of total energy and micronutrients. Intakes of micronutrients were calculated as absolute intake in the analyses, including vitamin A, thiamine, riboflavin, vitamin C, calcium, magnesium, phosphorus, iron, zinc, copper and selenium.

### 2.3. Assessment of Overweight and Obesity

Anthropometric measurements were conducted by well-trained health workers and nurses following the investigation manual. All the participants wore lightweight clothing and were without shoes when measuring height and weight, and these measurements were measured to the nearest 0.1 cm and 0.1 kg, respectively. BMI (body mass index) was calculated as weight in kilograms divided by height in meters squared (kg/m^2^). We used the body-mass-index-for-age Z-scores (BAZ) and standard deviations (SD) of 2006 World Health Organization (WHO) growth standards to define body weight status [17]. Children and adolescents’ obesity were defined as BAZ > +2 SD, overweight was defined as (BAZ > +1 SD), and underweight was defined as BAZ < −2 SD. In addition, between overweight and underweight was considered normal body weight.

### 2.4. Defining Micronutrient Deficiency and Intraindividual DBM

We applied estimated average requirement (EAR) as the cut-offs to assess dietary intake of the micronutrients in present study. EAR is the estimated nutrient intake level that would meet the requirement for half of the healthy individuals in a particular life stage and gender group. Micronutrient deficiency was defined as the subjects having the selected dietary micronutrients intake less than the EAR. Intraindividual DBM was defined as coexistence of overweight/obesity and dietary micronutrient intakes deficiencies.

### 2.5. Assessment of Covariates

The covariates we brought into the analysis were age, per capita annual family income, urbanization index (score) and residence region (urban and rural). According to the life stage in Chinese Dietary Reference Intakes, participants were divided into three groups (aged 6 to 10 years, 11 to 13 years and 14 to 17 years). The calculation of urbanization index based on 12 dimensions of the community level [18], and yearly income and urbanization index were divided into three groups (low, medium, and high) according to the annual per capita income level and urbanization index of subjects. Residency was classified into two categories (urban and rural).

### 2.6. Statistical Analysis

Mean daily dietary intake and standard error (SE) of the selected 11 micronutrients were calculated by body weight status in each subpopulation. According to the EAR of the Chinese Dietary Reference Intakes (2013) [19,20,21] (Table S1), the prevalence of Chinese children and adolescents aged 6 to 17 years not meeting the EAR were further estimated for selected micronutrients in each subpopulation separately by their body weight status. To describe the characteristics of the participants, categorical variables were expressed as frequencies and percentages, chi-square analysis was used to compare the differences; means and SEs were used to describe the distribution of micronutrient intake in different body weight categories. The generalized linear regression model was used to evaluate whether the average dietary intake of micronutrients varies among different body weight status through the incorporating of age into the model.

We conducted all statistical analyses using SAS version 9.4 (SAS Institute, Inc., Cary, NC, USA). All statistical tests were two-tailed and considered significant at *p* < 0.05.

## 3. Results

### 3.1. Participant Characteristics and Intraindividual DBM

As shown in Table 1, a total of 1555 children aged 6 to 17 years were included in the study, 51.83% of the participants were boys and 48.17% girls, 67.91% are rural subjects. The proportion of the subjects in different income levels and urbanization index levels is basically equal. 15.43% of the participants were overweight and 11.06% were obese, and the prevalence of underweight was 5.02%. Of the overweight subjects, there is a significant difference (*p* < 0.05) between urban and rural subjects, and the prevalence of overweight increased with the increase of yearly income and urbanization index. Of the obesity subjects, there is a significant difference (*p* < 0.05) between different gender and age groups, and the prevalence of obesity decreased with the increase of age. Intraindividual DBM was observed in 26.24% of children and adolescents, along with 4.95% of subjects affected by underweight and dietary micronutrient intake deficiency concurrently. The proportion of underweight and micronutrient deficiency in underweight subjects was 98.72%, and 99.03% overweight or obesity children and adolescents had dietary micronutrient deficiency in overweight/obesity subjects. (Table 1).

### 3.2. Disparity in Micronutrient Intake across Different Body Weight Status

Compared with normal weight boys, overweight or obese boys had higher dietary intake of riboflavin, vitamin C, phosphorus, magnesium, zinc and selenium, and the intake of vitamin C in obese boys was the highest. (Table 2). However, there was little difference in dietary micronutrient intakes in different body weight groups of girls, except the overweight girls had higher dietary intake of riboflavin, phosphorus and selenium than the normal weight counterparts. (Table 3).

### 3.3. Disparity in Micronutrient Deficiency Status across Different Body Weight Status

**Figure 1:**
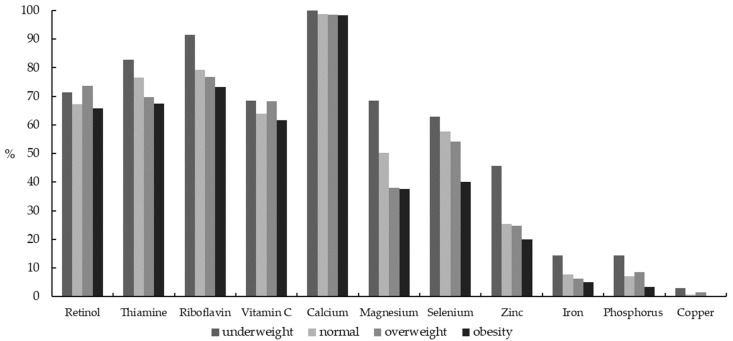
Percentage of Chinese boys aged 6–17 years with dietary micronutrient intakes below the estimated average requirements (EARs) by body weight status.

More than 50% boys of all body weight categories did not meet the EAR of retinol, thiamine, riboflavin, vitamin C, calcium, and calcium deficiency was nearly 100% in all body weight groups. Overweight boys had a high prevalence of dietary intake inadequacy of retinol (73.64% vs. 67.24%), vitamin C (68.22% vs. 63.98%), phosphorus (8.53% vs. 7.09%) and copper (1.55% vs. 0.57%) compared to normal weight boys. Compared with obese boys, overweight boys also had a high prevalence of dietary intake inadequacy of retinol (73.64% vs. 65.83%), thiamine (69.77% vs. 67.50%), riboflavin (76.74% vs. 73.33%), vitamin C (68.22% vs. 61.67%) and calcium (98.45% vs. 98.33%). Underweight boys had the highest prevalence of not meeting EAR for all the selected micronutrients. (Figure 1 and Figure S1)

**Figure 2:**
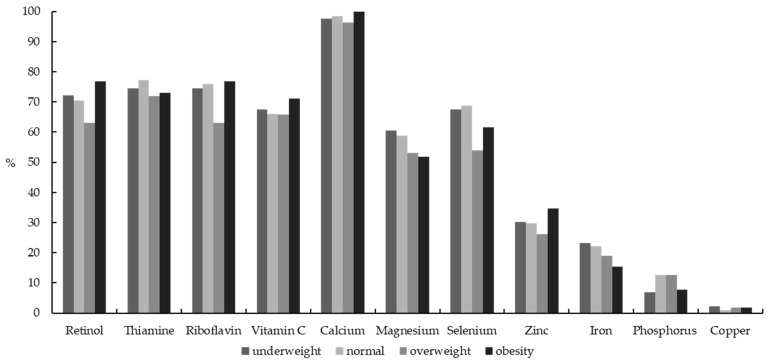
Percentage of Chinese girls aged 6–17 years with dietary micronutrient intakes below the estimated average requirements (EARs) by body weight status.

The prevalent characteristic of the selected micronutrients below the EAR was different in girls. Compared with normal girls, obese girls had a higher inadequacy of retinol (76.92% vs. 70.53%), riboflavin (76.92% vs. 76.06%), vitamin C (71.15% vs. 66.11%), calcium (100% vs. 98.53%), zinc (34.62% vs. 29.83%) and copper (1.92% vs. 0.92%). The dietary intake inadequacy of retinol, riboflavin, vitamin C, calcium and zinc below the EAR in obesity group was also noticeably the highest prevalence among these four groups in girls. (Figure 2 and Figure S2)

## 4. Discussion

This study explored the DBM among Chinese children and adolescents aged 6 to 17 years using the CHNS 2015, and examined whether daily dietary of micronutrient status varies by body weight categories. Compared with Chinese adults (39.58%) [22], the lower prevalence of DBM among children and adolescents (26.24%) appeared to be constrained by the low prevalence of overweight. With the increase number of overweight and obesity in China, an increase in prevalence of the DBM among children and adolescents at the individual level might happen. Previous studies have suggested the low dietary intakes of calcium [23], zinc [24], vitamin C [25] and other common micronutrients [12] in Chinese children and adolescents, but the prevalence of intake inadequacy of micronutrient was not calculated in those papers. In the present study, despite the dietary intake insufficiency existing in all the selected micronutrients among children and adolescents, the deficiency of vitamin A, B and C and calcium, magnesium and selenium were critical, and almost 100% participants were calcium deficient. The dietary patterns of Chinese children and adolescents have improved dramatically in the past 20 years [26], and the dietary fats, mean percentage of energy from total fat and intake of cholesterol had an increasing trend in Chinese aged 7–17 years [27], which may be related to the increasing prevalence of overweight and obesity. Cereals, potatoes, vegetables and fruits are rich in vitamins and minerals, and rice product, potato and vegetable intake in children and adolescents showed a downward trend [26], which may result in the insufficiency of micronutrients.

During the child and adolescent growth spurt, bone formation requires adequate calcium to form and lay down new bone properly. Long periods of adequate calcium intake in childhood increase bone mineral density and reduce osteopenia risk [28]. Ensuring adequate calcium intake during childhood and adolescence is critical to acquire good peak bone mass to prevent osteoporosis during older age [29]. In our study, dietary calcium inadequacy was almost 100%, which was the most prevalent deficiency of the selected micronutrients in Chinese aged 6–17 years. Previous studies reported that dietary calcium intake has been negatively related to body weight and/or body fat in children and adolescents [30,31]. Our results suggested that the prevalence of calcium intake insufficiency in obese girls was higher than in other body weight groups, but we did not explore the relationship between dietary calcium intake and overweight/obesity in present study. Dairy products as a good source of calcium, the low consumption of dairy products in Chinese children and adolescents may explain the low intake of dietary calcium [32]. Vegetables are a good source of dietary riboflavin and vitamin C, but the consumption rate of vegetables in Chinese children and adolescents was low, which can lead to the insufficient intake of riboflavin and vitamin C to a certain extent [26]. In addition, with the improvement of living standards, the whole grain consumption in China has gradually decreased [33], resulting in a high rate of thiamine insufficiency among children and adolescents. Other selected micronutrients insufficiency was also high though not as serious as calcium, which suggests unbalanced diets in Chinese children and adolescents. All the same, the dietary micronutrients intake insufficiency in children and adolescents was different from adults. With the increase of body weight, the risk of insufficient intake of micronutrients is relatively low, which was opposite of the results in adults [22]. Girls were more likely to have iron insufficiency than boys were, possibly because the cut-off point of the EAR of girls is higher than that of boys over 10 years old in Chinese Dietary Reference Intakes (2013). In addition, we observed dietary zinc inadequacy prevalence was particularly high in the obesity group of girls, but no similar results have been found in current studies. From another point of view, studies have shown that children take their parents’ dietary behavior, lifestyle and diet related attitude as examples [34], while peer influence increases during adolescence [35], which suggests parents’ and peers’ contributions to children and adolescents’ healthy diet should be examined as potential targets for intervention to reduce the intraindividual DBM. Therefore, improving the dietary structure and quality while promoting a positive food environment for children and adolescents are both important approaches to solve the intraindividual DBM.

Intraindividual DBM has different effects on the child’s development and cognitive performance. Childhood obesity negatively affects a child’s physical health, social and emotional well-being as well as obesity-related medical conditions [36]. Micronutrient deficiency has negative effects on physical and mental health [37], which also hinders the social progress and economic prosperity of a nation as a whole. The damage of micronutrient deficiency to the body is complex and diverse. When people suffer from multiple micronutrient deficiency, they are at higher risk for multiple impairments, such as iron, zinc deficiencies are associated with cognitive deficits among children [38]. The serious lack of retinol, thiamine, riboflavin, calcium, selenium, magnesium and zinc is probably because the poor dietary diversity and food variety in Chinese children [39]. The increase of consumption of sugary drinks and snacks with high energy and high fat may can explain the increasing prevalence of overweight/obesity in Chinese children and adolescents to some extent [40].

This study had some strengths. First, the CHNS are population-based surveys with large sample sizes. Second, the interviewers of the CHNS were professionally guided and experienced, which could assure the accuracy of anthropometric measurements and dietary estimates data. Third, few studies focused on the intraindividual DBM among Chinese aged 6–17 years, our results not only assessed the dietary micronutrient intakes but also combined with overweight/obesity to assess malnutrition. However, this study also had some limitations. First, our diet data was collected by three consecutive days of 24 h dietary recalls of children and adolescents. Despite the help of parents/guardians of children under 12 years during the diet investigation, the possibility of underestimating or overestimating the actual dietary intakes still exists. Second, the CHNS was sampled by family, the proportion of children and adolescents was relatively small in each family, so the sample size in present study was small. Third, the dietary supplement and medication of participants was not quantified in the CHNS, which may lead to some bias of the dietary micronutrient intake assessment. Fourth, the CHNS was conducted between August and November in the survey year, so the diet data may not reflect the seasonal differences, and the representativeness of foods is relatively weak. Moreover, the recall bias from self-reported diet may exist.

## 5. Conclusions

Under the background of the increasing prevalence of overweight and obesity, the present study revealed the DBM in children and adolescents is now pretty much the agenda for China, as well as the micronutrient intake insufficiency in each body weight group. Compared to the current reference values, the proportion of calcium, retinol, riboflavin, thiamin, vitamin C, selenium, zinc, and magnesium that did not meet the EAR among the selected micronutrients was high. Despite the gap on how to translate this evidence into effective actions, it is necessary to guide children as well as their parents to choose high-quality diets to reduce the risk of malnutrition in all its forms at all stages of the lifecycle.

## Figures and Tables

**Table 1:** Characteristics of study participants and prevalence of multiple forms of malnutrition, the China Health and Nutrition Survey (CHNS) 2015 ^1^.

Characteristics	Participants	Underweight	Overweight	Obesity	Underweight and Micronutrient Deficiency	Overweight/Obesity and Micronutrient Deficiency	UMD% ^2^	OMD% ^3^
Number of subjects	1555 (100.0)	78 (5.02)	240 (15.43)	172 (11.06)	77 (4.95)	408 (26.24)	77 (98.72)	408 (99.03)
Sex								
Boys	806 (51.83)	35 (4.34)	129 (16.00)	120 (14.89) ^c^	35 (4.34)	247 (30.65)	35 (100.00)	247 (99.20)
Girls	749 (48.17)	43 (5.74)	111 (14.82)	52 (6.94)	42 (5.61)	161 (21.50)	42 (97.67)	161 (98.77)
Age (year)								
6–10	858 (55.18)	41 (4.78)	128 (14.92)	111 (12.94) ^c, t^	40 (4.66)	236 (27.51)	40 (97.56)	236 (98.74)
11–13	417 (26.82)	23 (5.52)	75 (17.99)	39 (9.35)	23 (5.52)	114 (27.34)	23 (100.00)	114 (100.00)
14–17	280 (18.01)	14 (5.00)	37 (13.21)	22 (7.86)	14 (5.00)	58 (20.71)	14 (100.00)	58 (98.31)
Urban and rural								
Urban	499 (32.09)	21 (4.21)	94 (18.84) ^c^	63 (12.63)	21 (4.21)	154 (30.86)	21 (100.00)	154 (98.09)
Rural	1056 (67.91)	57 (5.40)	146 (13.83)	109 (10.32)	56 (5.30)	254 (24.05)	56 (98.25)	254 (99.61)
Yearly Income								
Low	519 (33.38)	34 (6.55) ^t^	65 (12.52) ^c, t^	44 (8.48)	34 (6.55)	109 (21.00)	34 (100.00)	109 (100.00)
Medium	518 (33.31)	24 (4.63)	72 (13.90)	60 (11.58)	24 (4.63)	131 (25.29)	24 (100.00)	131 (99.24)
High	518 (33.31)	20 (3.86)	103 (19.88)	68 (13.13)	19 (3.67)	168 (32.43)	19 (95.00)	168 (98.25)
Urbanicity index								
Low	521 (33.50)	27 (5.18)	61 (11.71) ^c, t^	47 (9.02)	27 (5.18)	108 (20.73)	27 (100.00)	108 (100.00)
Medium	516 (33.18)	27 (5.23)	89 (17.25)	62 (12.02)	27 (5.23)	150 (29.07)	27 (100.00)	150 (99.34)
High	518 (33.31)	24 (4.63)	90 (17.37)	63 (12.16)	23 (4.44)	150 (28.96)	23 (95.83)	150 (98.04)

^1^ All values are n (%). CHNS, China Health and Nutrition Survey; ^2^ proportion of underweight and micronutrient deficiency in underweight subjects; ^3^ proportion of overweight/obesity and micronutrient deficiency in overweight/obesity subjects; ^c^ showed a significant difference in the prevalence of multiple forms of malnutrition tested by Chi-square test (*p* < 0.05); ^t^ showed a significant difference in the prevalence of multiple forms of malnutrition tested by Chi square trend test (*p* < 0.05).

**Table 2:** Estimated daily micronutrient intakes from foods by category of body weight in boys aged 6–17 years, CHNS 2015 (*n* = 806) ^1^.

Micronutrient	Underweight	Normal	Overweight	Obesity
Retinol (µg RAE)	614.72 ± 148.56	487.93 ± 38.39	421.42 ± 77.23	434.25 ± 81.14
Thiamine (mg)	0.68 ± 0.06 ^a^	0.76 ± 0.02	0.81 ± 0.03	0.83 ± 0.03 ^b^
Riboflavin (mg)	0.64 ± 0.06 ^a^	0.71 ± 0.02 ^a, c^	0.78 ± 0.03 ^b^	0.76 ± 0.03
Vitamin C (mg)	57.35 ± 36.77	60.54 ± 9.50 ^a^	54.33 ± 19.12 ^a, b^	114.08 ± 19.84 ^c^
Calcium (mg)	308.82 ± 28.78	317.65 ± 7.44 ^a^	356.67 ± 14.96 ^b^	348.70 ± 15.52
Phosphorus (mg)	744.83 ± 48.75 ^a^	799.88 ± 12.60 ^a, d^	882.21 ± 25.34 ^b^	903.73 ± 26.30 ^b, c^
Magnesium (mg)	224.24 ± 15.84	220.69 ± 4.09 ^a^	236.94 ± 8.23	243.82 ± 8.55 ^b^
Iron (mg)	17.49 ± 1.78	19.15 ± 0.46	21.03 ± 0.93	20.36 ± 0.96
Zinc (mg)	8.12 ± 0.58 ^a^	9.02 ± 0.15 ^a, c, d^	9.83 ± 0.30 ^b^	9.70 ± 0.31 ^b, c^
Copper (mg)	1.46 ± 0.15	1.48 ± 0.04	1.49 ± 0.08	1.54 ± 0.08
Selenium (µg)	37.92 ± 3.71 ^a, d^	39.13 ± 0.96 ^a, c^	44.25 ± 1.93 ^b, d^	47.29 ± 2.00 ^b^

^1^ All values are means ± SEs; CHNS, China Health and Nutrition Survey; RAE, retinol activity equivalents; ^a, b, c, d^ *p* < 0.05 across body weight categories for boys calculated by using generalized linear models with adjustment for age, different letters indicate differences between the two groups, and the same letter indicates no difference between the two groups.

**Table 3:** Estimated daily micronutrient intakes from foods by category of body weight girls aged 6–17 years, CHNS 2015 (*n* = 749) ^1^.

Micronutrient	Underweight	Normal	Overweight	Obesity
Retinol (µg RAE)	313.19 ± 123.83	426.06 ± 34.84	479.71 ± 77.00	293.56 ± 113.08
Thiamine (mg)	0.67 ± 0.05	0.70 ± 0.01	0.73 ± 0.03	0.68 ± 0.05
Riboflavin (mg)	0.65 ± 0.05	0.67 ± 0.01 ^a^	0.75 ± 0.03 ^b^	0.64 ± 0.05
Vitamin C (mg)	52.88 ± 14.83	61.10 ± 4.17	63.44 ± 9.22	49.22 ± 13.54
Calcium (mg)	287.27 ± 29.32	306.36 ± 8.24	336.00 ± 18.23	328.41 ± 26.77
Phosphorus (mg)	734.00 ± 43.44	750.82 ± 12.22 ^a^	815.52 ± 27.01 ^b^	746.40 ± 39.67
Magnesium (mg)	199.69 ± 15.68	214.05 ± 4.41	214.36 ± 9.75	208.69 ± 14.32
Iron (mg)	16.54 ± 1.58	17.81 ± 0.44	18.34 ± 0.98	17.97 ± 1.44
Zinc (mg)	8.28 ± 0.53	8.51 ± 0.15	8.85 ± 0.33	7.92 ± 0.49
Copper (mg)	1.26 ± 0.12	1.39 ± 0.03	1.43 ± 0.08	1.26 ± 0.11
Selenium (µg)	35.17 ± 3.14	36.24 ± 0.88 ^a^	41.14 ± 1.95 ^b^	35.68 ± 2.87

^1^ All values are means ± SEs; CHNS, China Health and Nutrition Survey; RAE, retinol activity equivalents; ^a, b^ *p* < 0.05 across body weight categories for girls calculated by using generalized linear models with adjustment for age, different letters indicate differences between the two groups.

## Data Availability

Unavailable.

## Supplementary Materials

The following are available online at https://www.mdpi.com/article/10.3390/nu13093097/s1, Figure S1: Percentage of Chinese boys aged 6–17 years with dietary micronutrient intakes below the estimated average requirements (EARs) by body weight status; Figure S2: Percentage of Chinese girls aged 6–17 years with dietary micronutrient intakes below the estimated average requirements (EARs) by body weight status; Table S1. The Estimated Average Requirement (EAR) of selected micronutrients of Chinese children and adolescents.

## References

[1] Development Initiatives (2020). 2020 Global Nutrition Report: Action on Equity to End Malnutrition. https://globalnutritionreport.org/reports/global-nutrition-report-2020/.

[2] Popkin B.M., Corvalan C., Grummer-Strawn L.M. (2020). Dynamics of the double burden of malnutrition and the changing nutrition reality. Lancet.

[3] Barth-Jaeggi T., Zandberg L., Bahruddinov M., Kiefer S., Rahmarulloev S., Wyss K. (2020). Nutritional status of Tajik children and women: Transition towards a double burden of malnutrition. Matern. Child Nutr..

[4] Le Nguyen B.K., Le Thi H., Nguyen D.V., Tran T.N., Nguyen H.C., Thanh D.T., Deurenberg P., Khouw I. (2013). Double burden of undernutrition and overnutrition in Vietnam in 2011: Results of the SEANUTS study in 0.5–11-year-old children. Br. J. Nutr..

[5] Adair L.S., Gordon-Larsen P., Du S.F., Zhang B., Popkin B.M. (2014). The emergence of cardiometabolic disease risk in Chinese children and adults: Consequences of changes in diet, physical activity and obesity. Obes. Rev..

[6] Dearth-Wesley T., Wang H., Popkin B.M. (2008). Under- and overnutrition dynamics in Chinese children and adults (1991–2004). Eur. J. Clin. Nutr..

[7] Jia P., Xue H., Zhang J., Wang Y. (2017). Time trend and demographic and geographic disparities in childhood obesity prevalence in china-evidence from twenty years of longitudinal data. Int. J. Environ. Res. Public Health.

[8] Zhang J., Wang H., Wang Z., Du W., Su C., Zhang J., Jiang H., Jia X., Huang F., Ouyang Y. (2018). Prevalence and stabilizing trends in overweight and obesity among children and adolescents in China, 2011–2015. BMC Public Health.

[9] Guo Y., Yin X., Wu H., Chai X., Yang X. (2019). Trends in overweight and obesity among children and adolescents in China from 1991 to 2015: A meta-analysis. Int. J. Environ. Res. Public Health.

[10] Cui Z., Dibley M.J. (2012). Trends in dietary energy, fat, carbohydrate and protein intake in Chinese children and adolescents from 1991 to 2009. Br. J. Nutr..

[11] Wang Z.H., Zhai F.Y., Wang H.J., Zhang J.G., Du W.W., Su C., Zhang J., Jiang H.R., Zhang B. (2015). Secular trends in meat and seafood consumption patterns among Chinese adults, 1991–2011. Eur. J. Clin. Nutr..

[12] Wang H., Wang D., Ouyang Y., Huang F., Ding G., Zhang B. (2017). Do chinese children get enough micronutrients?. Nutrients.

[13] Mai T., Pham N.O., Tran T., Baker P., Gallegos D., Do T., van der Pols J.C., Jordan S.J. (2020). The double burden of malnutrition in Vietnamese school-aged children and adolescents: A rapid shift over a decade in Ho Chi Minh City. Eur. J. Clin. Nutr..

[14] Zhou S., Ye B., Fu P., Li S., Yuan P., Yang L., Zhan X., Chao F., Zhang S., Wang M.Q. (2020). Double burden of malnutrition: Examining the growth profile and coexistence of undernutrition, overweight, and obesity among school-aged children and adolescents in urban and rural counties in Henan Province, China. J. Obes..

[15] Popkin B.M., Du S., Zhai F., Zhang B. (2010). Cohort profile: The China health and nutrition survey—Monitoring and understanding socio-economic and health change in China, 1989–2011. Int. J. Epidemiol..

[16] Yang Y., Wang G., Pan X. (2009). China Food Composition.

[17] (2006). WHO Child Growth Standards: Length/Height-for Age Weight-for-Age, Weight-for-Length, Weight-for-Height and Body Mass Index-for-Age: Methods and Development.

[18] Jones-Smith J.C., Popkin B.M. (2010). Understanding community context and adult health changes in China: Development of an urbanicity scale. Soc. Sci. Med..

[19] (2017). Chinese Dietary Reference Intakes—Part 3: Trace Element.

[20] (2018). Chinese Dietary Reference Intakes—Part 4: Lipid-Soluble Vitamin.

[21] (2018). Chinese Dietary Reference Intakes—Part 5: Water-Soluble Vitamin.

[22] Huang Q., Wang L., Jiang H., Wang H., Zhang B., Zhang J., Jia X., Wang Z. (2020). Intra-individual double burden of malnutrition among adults in China: Evidence from the China Health and Nutrition Survey 2015. Nutrients.

[23] Zhang J., Wang H., Wang Z., Zhang J., Du W., Su C., Jiang H., Zhai F., Zhang B. (2013). Trend in dietary calcium intake among Chinese children and adolescents aged 4 to 17 years in nine provinces from 1991 to 2009. Zhonghua Liu Xing Bing Xue Za Zhi.

[24] Zhang J., Zhang B., Wang H., Wang Z., Zhang J., Zhai F. (2013). Nutrients intake trend of Chinese population in nine provinces from 1991 to 2009 (X) zinc intake trend of Chinese children aged 7–17 years. Acta Nutr..

[25] Wang Z., Zhang B., Wang H., Zhang J., Du W., Su C., Zhang J., Zhai F. (2012). Trend in dietary vitamin C intake among Chinese children and adolescents between 1991 and 2009. Zhonghua Yu Fang Yi Xue Za Zhi.

[26] Wang X., Su C., Ouyang Y., Li W., Zhang B., Wang H. (2016). Trends of the Chinese in dietary pattern of children and adolescents in communities at different urbanization levels. Acta Nutr..

[27] Su C., Wang H., Wang Z., Zhang J., Du W., Zhang J., Zhai F., Zhang B. (2012). Current status and trends of both dietary fat and cholesterol intake among Chinese children and adolescents aged 7 to 17 years old in 9 provinces of China, from 1991 to 2009. Zhonghua Liu Xing Bing.

[28] Closa-Monasterolo R., Zaragoza-Jordana M., Ferre N., Luque V., Grote V., Koletzko B., Verduci E., Vecchi F., Escribano J. (2018). Adequate calcium intake during long periods improves bone mineral density in healthy children. Data from the Childhood Obesity Project. Clin. Nutr..

[29] Pan K., Zhang C., Yao X., Zhu Z. (2020). Association between dietary calcium intake and BMD in children and adolescents. Endocr. Connect..

[30] Skinner J.D., Bounds W., Carruth B.R., Ziegler P. (2003). Longitudinal calcium intake is negatively related to children’s body fat indexes. J. Am. Diet Assoc..

[31] Davies K.M., Heaney R.P., Recker R.R., Lappe J.M., Barger-Lux M.J., Rafferty K., Hinders S. (2000). Calcium intake and body weight. J. Clin. Endocrinol. Metab..

[32] He Y., Yang X., Xia J., Zhao L., Yang Y. (2016). Consumption of meat and dairy products in China: A review. Proc. Nutr. Soc..

[33] He Y., Fang Y., Yang Y. (2019). Whole grains and mixed beans intake among Chinese adolescents aged 6–17 years. Acta Nutr..

[34] Scaglioni S., De Cosmi V., Ciappolino V., Parazzini F., Brambilla P., Agostoni C. (2018). Factors influencing children’s eating behaviours. Nutrients.

[35] Chung S.J., Ersig A.L., McCarthy A.M. (2017). The influence of peers on diet and exercise among adolescents: A systematic review. J. Pediatr. Nurs..

[36] Sahoo K., Sahoo B., Choudhury A.K., Sofi N.Y., Kumar R., Bhadoria A.S. (2015). Childhood obesity: Causes and consequences. J. Fam. Med. Prim. Care.

[37] Black R.E., Allen L.H., Bhutta Z.A., Caulfield L.E., de Onis M., Ezzati M., Mathers C., Rivera J. (2008). Maternal and child undernutrition: Global and regional exposures and health consequences. Lancet.

[38] Black M.M. (2003). Micronutrient deficiencies and cognitive functioning. J. Nutr..

[39] Meng L., Wang Y., Li T., Loo-Bouwman C., Zhang Y., Man-Yau S.I. (2018). Dietary diversity and food variety in Chinese children aged 3–17 years: Are they negatively associated with dietary micronutrient inadequacy?. Nutrients.

[40] Du W., Wang H., Wang D., Su C., Zhang J., Ouyang Y., Jia X., Huang F., Zhang B. (2016). Meal and snack consumption among Chinese children and adolescents in twelve provinces. Wei Sheng Yan Jiu.

