# Supplementary material for: Intraindividual Double Burden of Malnutrition in Chinese Children and Adolescents Aged 6–17 Years: Evidence from the China Health and Nutrition Survey 2015
Source: Nutrients. 2021 Sep 3;13(9):3097. doi: 10.3390/nu13093097 (PMC8469495; doi:10.3390/nu13093097)
Supplement: Supplementary file 1 [file nutrients-13-03097-s001.zip › nutrients-1324064-SI.pdf]

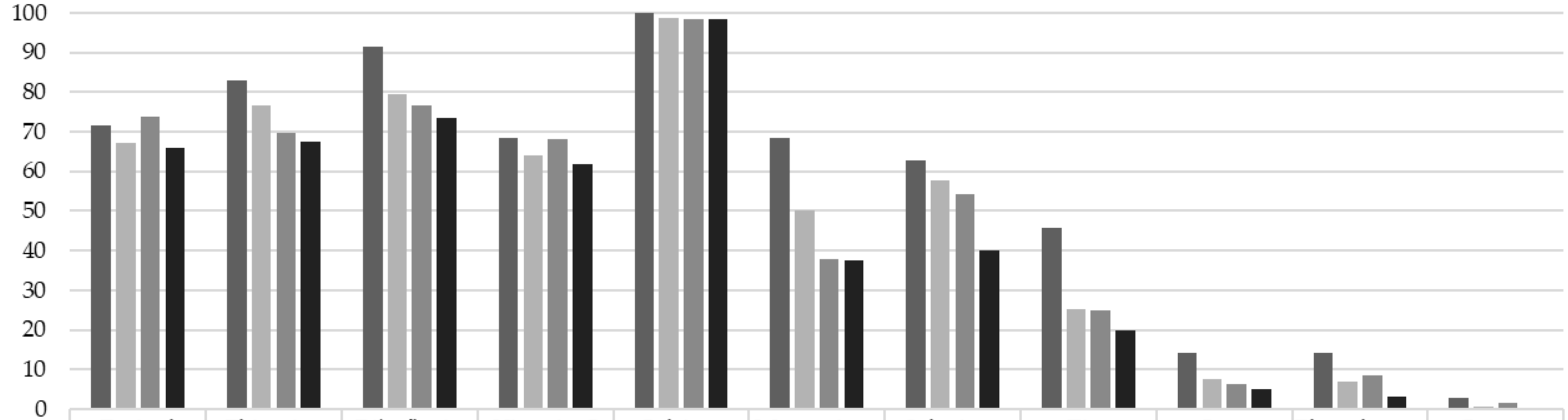


| | Retinol | Thiamine | Riboflavin | Vitamin C | Calcium | Magnesium | Selenium | Zinc | Iron | Phosphorus | Copper |
|---|---|---|---|---|---|---|---|---|---|---|---|
| underweight | 71.43 | 82.86 | 91.43 | 68.57 | 100 | 68.57 | 62.86 | 45.71 | 14.29 | 14.29 | 2.86 |
| normal | 67.24 | 76.63 | 79.31 | 63.98 | 98.66 | 50.19 | 57.66 | 25.29 | 7.66 | 7.09 | 0.57 |
| overweight | 73.64 | 69.77 | 76.74 | 68.22 | 98.45 | 37.98 | 54.26 | 24.81 | 6.2 | 8.53 | 1.55 |
| obesity | 65.83 | 67.5 | 73.33 | 61.67 | 98.33 | 37.5 | 40 | 20 | 5 | 3.33 | 0 |

underweight normal overweight obesity

**Figure S1.** Percentage of Chinese boys aged 7–17 years with dietary micronutrient intakes below the estimated average requirements (EARs) by body weight status

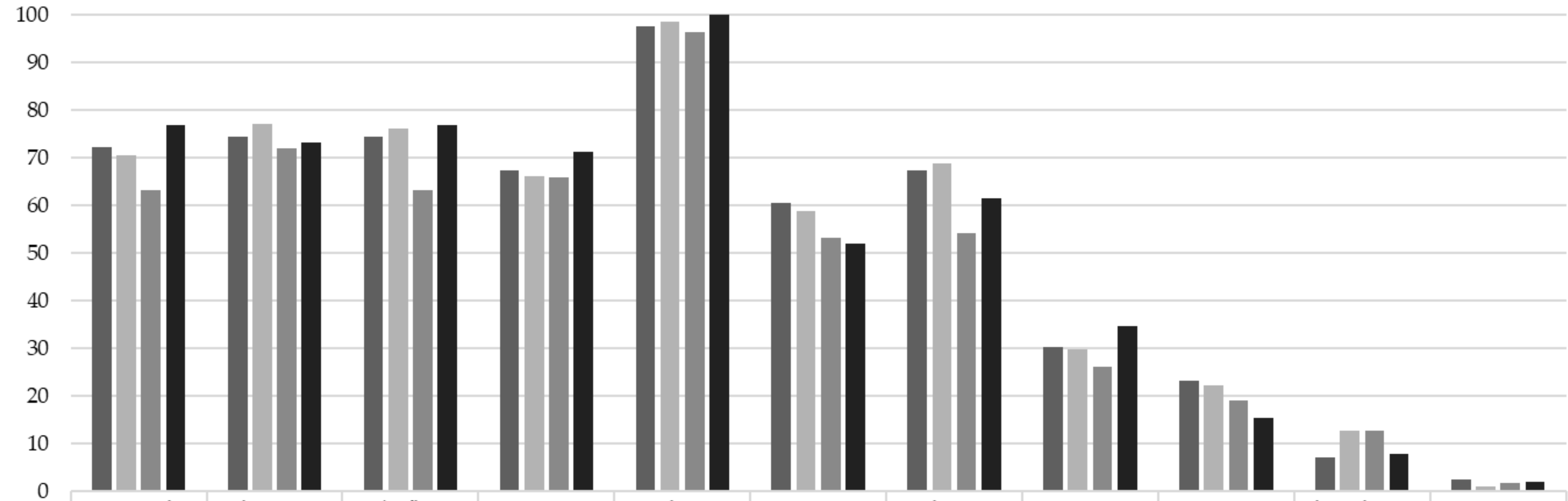


| | Retinol | Thiamine | Riboflavin | Vitamin C | Calcium | Magnesium | Selenium | Zinc | Iron | Phosphorus | Copper |
|---|---|---|---|---|---|---|---|---|---|---|---|
| underweight | 72.09 | 74.42 | 74.42 | 67.44 | 97.67 | 60.47 | 67.44 | 30.23 | 23.26 | 6.98 | 2.33 |
| normal | 70.53 | 77.16 | 76.06 | 66.11 | 98.53 | 58.75 | 68.88 | 29.83 | 22.1 | 12.71 | 0.92 |
| overweight | 63.06 | 72.07 | 63.06 | 65.77 | 96.4 | 53.15 | 54.05 | 26.13 | 18.92 | 12.61 | 1.8 |
| obesity | 76.92 | 73.08 | 76.92 | 71.15 | 100 | 51.92 | 61.54 | 34.62 | 15.38 | 7.69 | 1.92 |

underweight normal overweight obesity

**Figure S2.** Percentage of Chinese girls aged 7–17 years with dietary micronutrient intakes below the estimated average requirements (EARs) by body weight status

**Table S1.** The Estimated Average Requirement (EAR) of selected micronutrients

| years | Retinol (μg RAE/d) | | Thiamine (mg/d) | | Riboflavin (mg/d) | | Vitamin C (mg/d) | Calcium (mg/d) | Phosphorus (mg/d) | Magnesium (mg/d) | Iron (mg/d) | | Zinc (mg/d) | | Copper (mg/d) | Selenium (μg/d) |
|---|---|---|---|---|---|---|---|---|---|---|---|---|---|---|---|---|
| | M[1] | F | M | F | M | F | | | | | M | F | M | F | | |
| 7-10 | 360 | | 0.8 | | 0.8 | | 55 | 800 | 400 | 180 | 10 | | 5.9 | | 0.4 | 35 |
| 11-13 | 480 | 450 | 1.1 | 1.0 | 1.1 | 0.9 | 75 | 1000 | 540 | 250 | 11 | 14 | 8.2 | 7.6 | 0.55 | 45 |
| 14-17 | 590 | 450 | 1.3 | 1.1 | 1.3 | 1.0 | 85 | 800 | 590 | 270 | 12 | 14 | 9.7 | 6.9 | 0.6 | 50 |

[1] Some of micronutrients requirement differs by gender.
